# Berberrubine protects against cisplatin-induced ototoxicity by promoting folate biosynthesis

**DOI:** 10.3389/fphar.2024.1496917

**Published:** 2025-01-09

**Authors:** Zhuang Miao, Danyang Chang, Xiaodong Du, Changling Sun

**Affiliations:** Department of Otolaryngology-Head and Neck Surgery, Affiliated Hospital of Jiangnan University, Wuxi, Jiangsu, China

**Keywords:** berberrubine, cisplatin, ototoxicity, hair cells, folate, oxidative stress

## Abstract

**Objective:**

This research investigated the possible shielding properties of BB (Berberrubine) against the harmful auditory effects of cisplatin, preliminarily delving into the underlying mechanisms responsible for this protection.

**Methods:**

HEI-OC1 cell viability was determined using a Cell Counting Kit-8 (CCK-8). The impact of BB on cochlear hair cells was studied through *in vitro* cochlear explants culture. Apoptosis levels were measured through Annexin V-PI, Cleaved Caspase-3, and TUNEL staining. The level of ROS (reactive oxygen species) was measured through the application of DCFH-DA, MitoSOX, and JC-1 fluorescent dyes for staining. Immunofluorescence analysis of cochlear samples from mice was conducted to quantify the hair cell count, and concurrently, ABR (Auditory Brainstem Response) testing was utilized to evaluate auditory function. The mechanism of action of BB was explored using RNA-Seq and qRT-PCR analysis.

**Results:**

BB significantly improved cell survival rates under cisplatin treatment, reduced levels of apoptotic markers (TUNEL, Cleaved Caspase-3, Annexin V-PI), decreased ROS and MitoSOX levels, and improved JC-1 signals in both HEI-OC1 cells and cochlear hair cells in cochlear explants culture. Animal studies demonstrated that treatment with BB enhanced the survival of cochlear hair cells, reduced hearing impairment caused by cisplatin in mice. RNA-seq and qRT-PCR analysis revealed that BB influenced the expression levels of multiple genes (*Ccnd2, Reln, Pgf, Mylk3, Ppplr12c, Thbsl*), by promoting folate biosynthesis for hearing protection.

**Conclusion:**

Our findings suggest that BB protects against cisplatin-induced hearing damage by enhancing folate biosynthesis, decreasing intracellular ROS levels, and inhibiting apoptosis.

## 1 Introduction

Hearing loss is a widespread global concern that profoundly affects the quality of life. Numerous factors can contribute to it, including genetics, aging, noise exposure, diseases, drug-induced damage, and trauma, among others (Albera et al., 2010; Gong et al., 2018; Liu and Yan, 2007). Among drug-induced cases, hearing loss caused by ototoxicity from cisplatin is commonly observed in clinical environments. Cisplatin, a chemotherapy agent utilized extensively, is potent against several types of cancers such as ovarian, prostate, testicular, lung, nasopharyngeal, esophageal, lymphoma, head and neck squamous cell carcinoma, and osteogenic sarcoma (Dasari and Tchounwou, 2014; Makovec, 2019; Romani, 2022; Tang et al., 2024). Although effective, the prevalence of side effects including kidney damage, nerve toxicity, and hearing impairment restricts its widespread use (Boulikas and Vougiouka, 2003; Crona et al., 2017; Fetoni et al., 2022). Techniques to prevent and treat cisplatin-induced nephrotoxicity and neurotoxicity are currently available; however, effective treatments for ototoxicity are still limited. Research indicates that ototoxicity from cisplatin is characterized by progressive, dose-dependent, and bilateral auditory damage (Callejo et al., 2017; Callejo et al., 2015; Guidotti et al., 2023). The suspected mechanisms involve oxidative stress, inflammatory response, apoptosis, and autophagy, which impair critical physiological components such as hair cells, stria vascularis, and spiral ganglia, although the precise details remain unclear (Guo et al., 2018; Kros and Steyger, 2019; Li Y. et al., 2023; Steyger, 2021). Therefore, it is crucial to identify medications or interventions that can offer protection,and investigate the underlying molecular pathways of cisplatin-related hearing damage.

**FIGURE 1:**
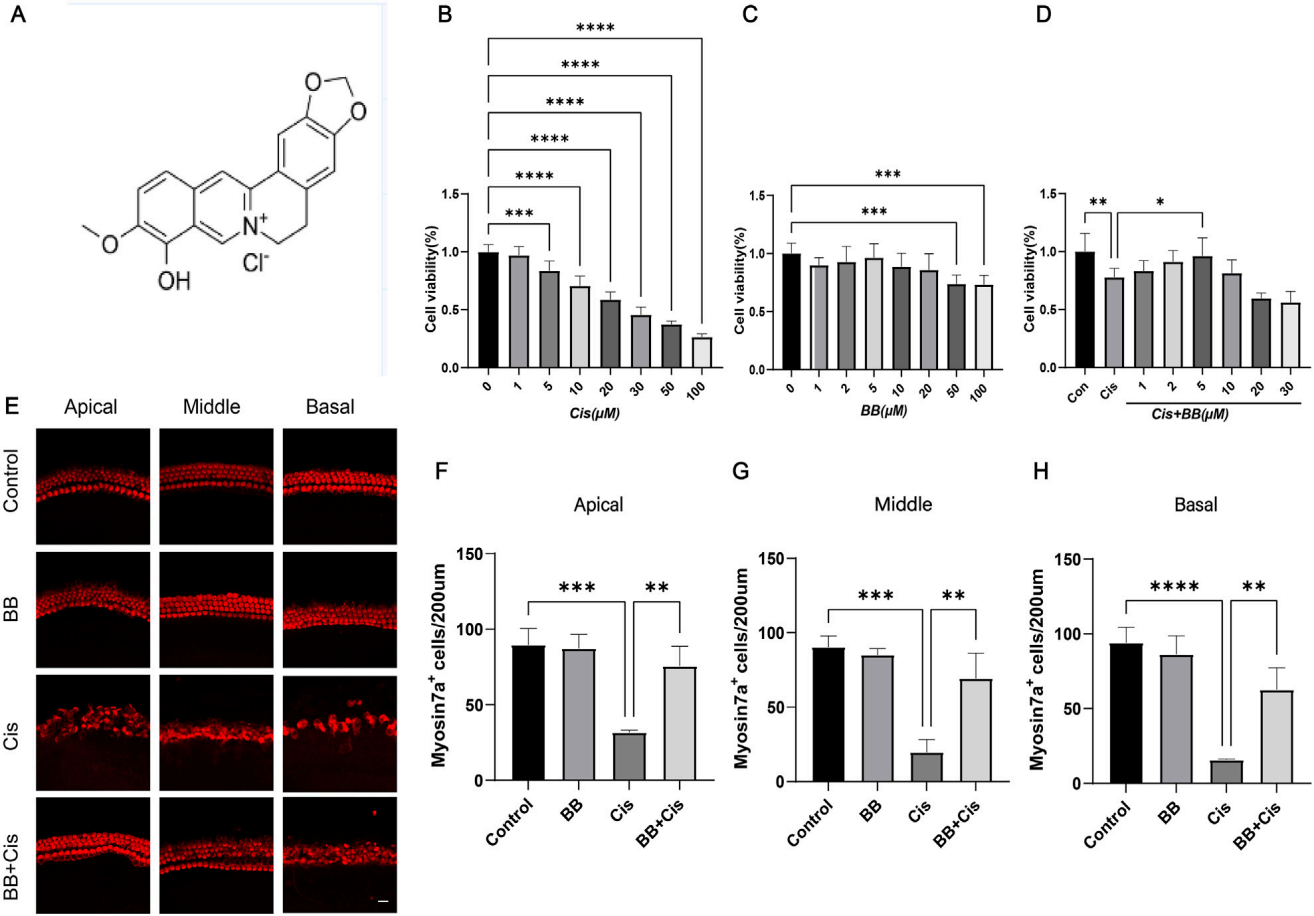
BB enhances cell survival in the exposure of cisplatin. **(A)** Chemical structure of BB. **(B)** CCK-8 assay shows cisplatin toxicity in HEI-OC1 cells at various concentrations. **(C)** CCK-8 assay indicates BB toxicity at different dosages. **(D)** CCK-8 assay reveals BB’s protective effect on cisplatin-treated HEI-OC1 cells. **(E)** Immunofluorescence of cochlear hair cells labeled with myosin 7a at the cochlea’s apex, midsection, and base. **(F–H)** Quantitative assessment of hair cell counts in the cochlea’s apex, midsection, and base. *P < 0.05, **P < 0.01, ***P < 0.001, ****P < 0.0001. BB: Berberrubine; Cis: Cisplatin.

Berberine exhibits potent pharmacological properties. Berberine exhibits antioxidant, anti-inflammatory, antimicrobial, antitumor, and neuroprotective activities (Jin et al., 2016; Song et al., 2020; Wang et al., 2017). Despite these potent effects, the relatively low concentration of berberine found in circulation seems to contrast with its extensive pharmacological influence. Conversely, the substantial presence of Berberrubine (BB, Figure 1A) in the bloodstream, as the predominant metabolite of berberine, suggests that it may significantly contribute to the overall therapeutic impact attributed to berberine. Furthermore, studies have indicated that BB possesses a beneficial ameliorative effect on diseases related to inflammatory-oxidative stress. Given that inflammatory response and oxidative stress are key mechanisms in cisplatin ototoxicity, this suggests that BB theoretically holds potential in preventing cisplatin-induced hearing damage (Li et al., 2010; Sun et al., 2021; Wang et al., 2020). In this research, we examined BB’s protective role against cisplatin-induced ototoxicity *via* cellular experiments, cochlear explants culture, and *in vivo* animal studies. Additionally, we investigated the underlying mechanisms of its action, which may offer a potential therapy for cisplatin-induced ototoxicity.

## 2 Materials and methods

### 2.1 Cell culture

HEI-OC1 line were cultivated in 10-cm circular dishes for cell culture, using high-glucose medium (Gibco, 11965092, United States) supplemented with 10% fetal bovine serum (Gibco, A5670701, United States) and 0.1% ampicillin (Beyotime, ST008, China). The cell cultures were maintained at a temperature of 33°C in a controlled atmosphere with 10% CO2 concentration. Due to the unique properties of HEI-OC1 cells, their metabolic state is more stable under 33 °C culture conditions, which can better promote cell proliferation and reduce cell differentiation. Cells were passaged using a solution of 0.25% trypsin with EDTA (Gibco, 25200056, United States) when the cell population in the dish reached approximately 80%–90%.

### 2.2 Cochlear explants culture

Six mice were sterilized using 75% alcohol before undergoing dissecting to extract the cochlea. The isolated cochlea was then immersed in a 6 cm dissecting dish containing Hank’s Balanced Salt Solution (HBSS) (Beyotime, C0219, China). Using fine tweezers on a sterile surface, the spiral ligament, cochlear implant, and vascular structure were meticulously removed to isolate the cochlear basement membrane. This membrane was subsequently laid flat on a Cell-Tak-coated circular slide (Corning, 354,240, United States), which was then transferred to a four-hole dish with a six-cm diameter. The cochleas, numbering a total of twelve, were divided into separate experimental groups, control, BB-only, cisplatin-only, and BB combined with cisplatin groups. Each subgroup contained three basement membranes. These specimens were cultured in 3 mL of F12 high-glucose medium (Gibco, 11330–032, United States) along with B27 (Thermo Fisher, 17504044, United States), N2 (Thermo Fisher, 17502001, United States), and ampicillin, and were kept in a 5% carbon dioxide incubator at 37°C. After the attachment phase, Berberrubine (TargetMol, 15401–69–1, United States) was added to both the BB-only and the BB combined with cisplatin groups, achieving a final concentration of 5 μM and subsequently pre-incubated for 12 h. Subsequently, cisplatin was introduced to the groups cisplatin-only and BB combined with cisplatin groups, reaching a final concentration of 50 μM, after which the incubation continued for another 24 h.

### 2.3 Mouse models

Four-week-old male C57BL/6 mice were procured from Nanjing Qinglongshan Animal Co., Ltd., China. The animal studies conducted in strict compliance with the ethical standards of Southeast University, aligning with the national laws and regulations for animal experimentation. A total of twenty-four mice aged P28 were divided into four groups: control, BB gavage, cisplatin injury, and combined BB gavage with cisplatin injury. In the first week, the BB gavage groups and combined BB gavage with cisplatin injury groups received 20 mg/kg of BB (Yuanye, 15401–69–1, China) *via* gavage. The control and cisplatin injury groups were administered 0.9% saline orally. Moving into the second week, the gavage regimen continued for all groups, with the cisplatin-injured and combined BB gavage with cisplatin injury groups receiving an additional daily intraperitoneal injection of 4 mg/kg cisplatin. Similarly, the control group and the BB gavage group received injections of an equal amount of normal saline solution. Following the treatment period, the mice underwent ABR testing and hair cell counting.

### 2.4 Cell viability assay

The measurement of how alive the cells was done using the Cell Counting Kit-8 (CCK-8) (MCE, HY-K0301, United States). To begin with, the HEI-OC1 cells that were grown well were exposed to a 0.25% trypsin solution, and the enzymatic action was stopped by supplementing with an equivalent amount of growth medium. Subsequently, the cells were transferred to 15 mL centrifuge tubes for spinning at a speed of 1,000 rpm over a period of 5 min. The concentration of cells was determined with a cell counting chamber, followed by their seeding into 96-well plates at a density of 5,000 cells per well. Each well was categorized into different groups based on drug concentration, with 6 replicates wells in each group. Post drug treatment, CCK-8 reagent diluted in serum-free culture medium was added to each well and incubated for 1 h. A microplate reader was used to measure the absorbance at 450 nm, which was then utilized to compute the percentage of viable cells based on the obtained optical density readings.

### 2.5 TUNEL staining

To perform TUNEL staining, the 5 X equilibrium solution was first diluted to 1 X. Following dilution, the sample was placed in a four-well dish, and 1X equilibrium solution was added for equilibration for 30 min. Throughout this timeframe, the TUNEL reaction system (Beyotime, C1086, China) was prepared by combining ddH_2_O, equilibrium solution, a fluorescent dye, and terminal deoxynucleotidyl transferase (TDT). After the equilibration process was finished, the balanced solution was removed, and 50 μL of the TUNEL reagent was added to each well of the 4-well dish. Following that step, the dish was placed in an incubator maintained at a temperature of 37°C for a duration of 1 hour, ensuring it was kept in the dark to prevent light exposure. Upon completion of the incubation period, the TUNEL staining solution was decanted, and DAPI staining solution (from Beyotime, C1002, China) was applied. Subsequently, the samples were subjected to a further incubation period of 1 hour at ambient temperature. Once the incubation was complete, the medium was aspirated off, and the samples underwent three successive rinses using PBS (Beyotime, C0221A, China) for 5 min per wash. The samples were subsequently sealed using nail polish and analyzed using a confocal microscope. Should immunofluorescence staining be necessary, the samples would undergo the appropriate immunofluorescence staining procedure.

### 2.6 Annexin V-PI staining

For Annexin V-PI staining, initially the drug-treated cell samples underwent two washes with pre-cooled PBS. Subsequently, the Annexin V and PI Staining Solution, which was prepared using Binding Buffer (Beyotime, C1062S, China), was introduced to the cell samples. Using a pipette, Annexin V and PI Staining Solution was gently agitated to ensure thorough interaction with the cells. The cells were incubated for 10 min at ambient temperature. The setting was shielded from light. After incubation, the staining solution was aspirated or discarded and DAPI Staining Solution was applied for an additional 10-minute period. Following the DAPI staining procedure, the samples underwent three consecutive washes using PBS to remove any excess stain. To conclude the process, the samples were transferred onto microscope slides, and covered with coverslips, and then analyzed under a confocal scanning microscope.

### 2.7 ROS staining

To conduct ROS staining, had the culture medium removed. DCFH-DA (Beyotime, S0033M, China) was prepared by diluting it in a medium devoid of serum to a concentration ratio of 1:1,000. The diluted DCFH-DA solution was then applied to ensure full coverage of the cells. Following the preparation, The cells were placed in an incubator set to 37°C for 20 min. After the incubation, the cells underwent three washes with serum-free DMEM. This was followed by the removal of the staining solution and incubating the cells with DAPI staining for 10 min at room temperature. After the incubation, the cells underwent three rounds of washing using PBS. The samples were mounted onto slides with coverslips in place and then examined using a confocal microscope for detailed observation.

### 2.8 MitoSOX staining

For MitoSOX staining, begin by removing the drug-treated samples from the culture medium. Prepare a MitoSOX staining solution (Beyotime, S0061S, China), dilute it to a concentration of 5 μM with HBSS. Apply the prepared MitoSOX solution to the samples, then place them in an incubator set to 37°C for a duration of 10 min, to ensure taking care to protect them from light exposure. Following incubation, carefully remove the staining solution and wash the samples three times with pre-warmed HBSS at 37°C, each wash lasting for 5 min. After the washing process, mount the cells onto slides and incubate them with DAPI staining solution at room temperature for 10 min, again protecting them from light. Subsequent washing of the samples should be done three times with PBS. To conclude, the cell samples onto microscope slides secure with coverslips, and then proceed to examine them under a confocal microscope. If immunofluorescence staining is necessary, follow the relevant immunofluorescence staining protocol.

### 2.9 JC-1 mitochondrial membrane potential staining

To stain mitochondrial membrane potential, The working solution of JC-1 was diluted using ultra-pure water initially. Removal of the culture medium from the treated cell samples took place, followed by a single wash with PBS. Subsequently, the working solution of JC-1 (Beyotime, C2006, China) was combined with the culture medium and applied to the cell samples. The cells were then incubated at 37°C for 20 min. The JC-1 staining buffer was prepared using ultra-pure water and stored on ice. After the 20-minutes incubation, elimination of the staining solution and double washing of samples with the staining buffer took place. Lastly, the samples were observed using a confocal microscope.

### 2.10 Immunofluorescence analysis

HEI-OC1 cells, cochlear basement membrane, and cochlea samples underwent fixation in 4% paraformaldehyde. For the adult rat cochlea samples decalcification with EDTA solution was carried out prior to fixation. The cochlear basement membrane was then carefully dissected into three slices representing different turns (apical, middle, and basal) under a microscope post decalcification and fixation. Following the sample processing, the samples were permeabilized with PBST for 5 min, repeated three times. Subsequently, the samples were soaked with an immunofluorescence sealing solution for an hour. Once the blocking step was complete, the primary antibody specific for Myosin7a was introduced to the samples, which were then incubated at a temperature of 4°C in a refrigerator environment protected from light for an extended period overnight. On the following day, the primary antibody solution was aspirated off, the samples were washed extensively with PBST in three rounds of 5-minute intervals. Subsequent steps included the addition of the secondary antibody (goat anti-mouse AlexaFluor Plus 555) and DAPI, followed by an incubation period of 1 h at room temperature in the dark. Subsequently, the secondary antibody solution was eliminated, followed by a rigorous washing of the samples using PBST, which was done in triplicate for durations of 5 minutes per wash. When prepared, the samples were meticulously mounted onto DAKO-brand slides, topped with coverslips, and secured with a layer of clear nail polish to prevent leakage. The slides were left at room temperature for half an hour to allow the nail polish to dry before observation under a confocal microscope. The experiment utilized various primary and secondary antibodies as well as dyes such as goat anti-rabbit Alexa Fluor Plus 488, goat anti-rabbit Alexa Fluor Plus 555, goat anti-mouse AlexaFluor Plus 555 and phalloidin, in addition to DAPI staining solution. This meticulous process ensures the precise visualization and analysis of the cochlear structures and components in the samples, contributing valuable insights to the study of auditory system biology.

### 2.11 Auditory Brainstem Response (ABR) threshold

Mice were administered an isobarbital solution at a dosage rate of 10 mg/kg of body weight, rendered unconscious and then placed on a warming pad maintained at a temperature of 37°C. Judging the effectiveness of anesthesia by finger pinching reflex, if there is no response when pinching the toes of mice, it indicates that anesthesia is complete. Following anesthesia, ABR thresholds were recorded using a TDT system (Tucker Davies Technologies, Gainesville, FL, United States) at frequencies of 4, 8, 12, 16, 24, and 32 kHz.

### 2.12 RNA sequencing (RNA-Seq)

The HEI-OC1 cells were distributed into two groups and cultured in six-well plates, designating three wells per group for the experiment. One of the groups served as the control, while the other was subjected to BB treatment. Once the cells had adhered, the experimental group received BB at a concentration of 5 μM, which was maintained for a period of 24 h. Following the treatment, RNA was harvested from the cells in each well and then proceeded to RNA sequencing, conducted by Lianchuan Biotechnology Co., Ltd., China. following successful RNA quality control checks.

### 2.13 Quantitative real-time PCR (qRT-PCR)

Total RNA was extracted from lysed cells and reverse-transcribed into cDNA using a cDNA synthesis kit (Vazyme, RC112-01, China). The qRT-PCR reaction was set up with primers and a qRT-PCR master mix (Vazyme, Q511-02, China), The qRT-PCR program started with an initial denaturation at 95°C for 5 min, then 40 cycles of alternating between 95°C for 10 s and 60°C for 30 s. Following the amplification cycles, a melting curve analysis was conducted, which included steps at 95°C for 15 s, 60°C for a minute, and a final step at 95°C for another 15 s. The collected data were analyzed through the comparative Ct (cycle threshold) method, utilizing GAPDH as a housekeeping gene for normalization.

### 2.14 Statistical analysis

The data are expressed as mean values with their accompanying standard deviations (SD). For data that follows a normal distribution, a Student’s t-test was employed to assess the differences between two-group comparisons, while analysis of variance (ANOVA) followed by the Dunnett’s test was used for the comparison of three or more groups. For data that does not conform to a normal distribution, we use logarithmic transformation to transform it into data that conforms to a normal distribution, and then use the above statistical methods for analysis. Statistical analyses were conducted using Microsoft Excel and GraphPad Prism 9, with results considered statistically significant for *P* < 0.05.

## 3 Results

### 3.1 BB enhances cell survival in the exposure of cisplatin

A range of cisplatin concentrations, spanning from 0 to 100 μM in increments of 1 μM, 5 μM, 10 μM, 20 μM, 30 μM, 50 μM and 100 μM was applied to HEI-OC1 cells for exposure. The study’s findings indicated that cisplatin exhibited a lethal impact on approximately 50% of the HEI-OC1 cells within the concentration range of 20–30 μM (Figure 1B). Therefore, we chose 30 μM cisplatin for further investigation. To establish the optimal concentration of BB for treating HEI-OC1 cells before cisplatin treatment, varying doses of BB (1, 2, 5, 10, 20, 50, and 100 μM) were tested. The CCK-8 assay showed no toxic effects at cisplatin concentrations from 1 to 20 μM (Figure 1C). When HEI-OC1 cells were pre-incubated with BB concentrations ranging from 1 to 30 μM for 12 h, followed by concurrent treatment with 30 μM cisplatin and BB for an additional 24 h, it was found that a 5 μM of BB was effective in mitigating the damage caused by cisplatin to the cells (Figure 1D). Additionally, in explants culture models, pre-treatment with 5 μM BB alleviated the hair cell damage caused by cisplatin (Figures 1E–H). In summary, BB alleviates cisplatin-induced cell injury, enhancing cell survival in the exposure of cisplatin.

### 3.2 BB shield mice from hearing impairment caused by cisplatin exposure

**FIGURE 2:**
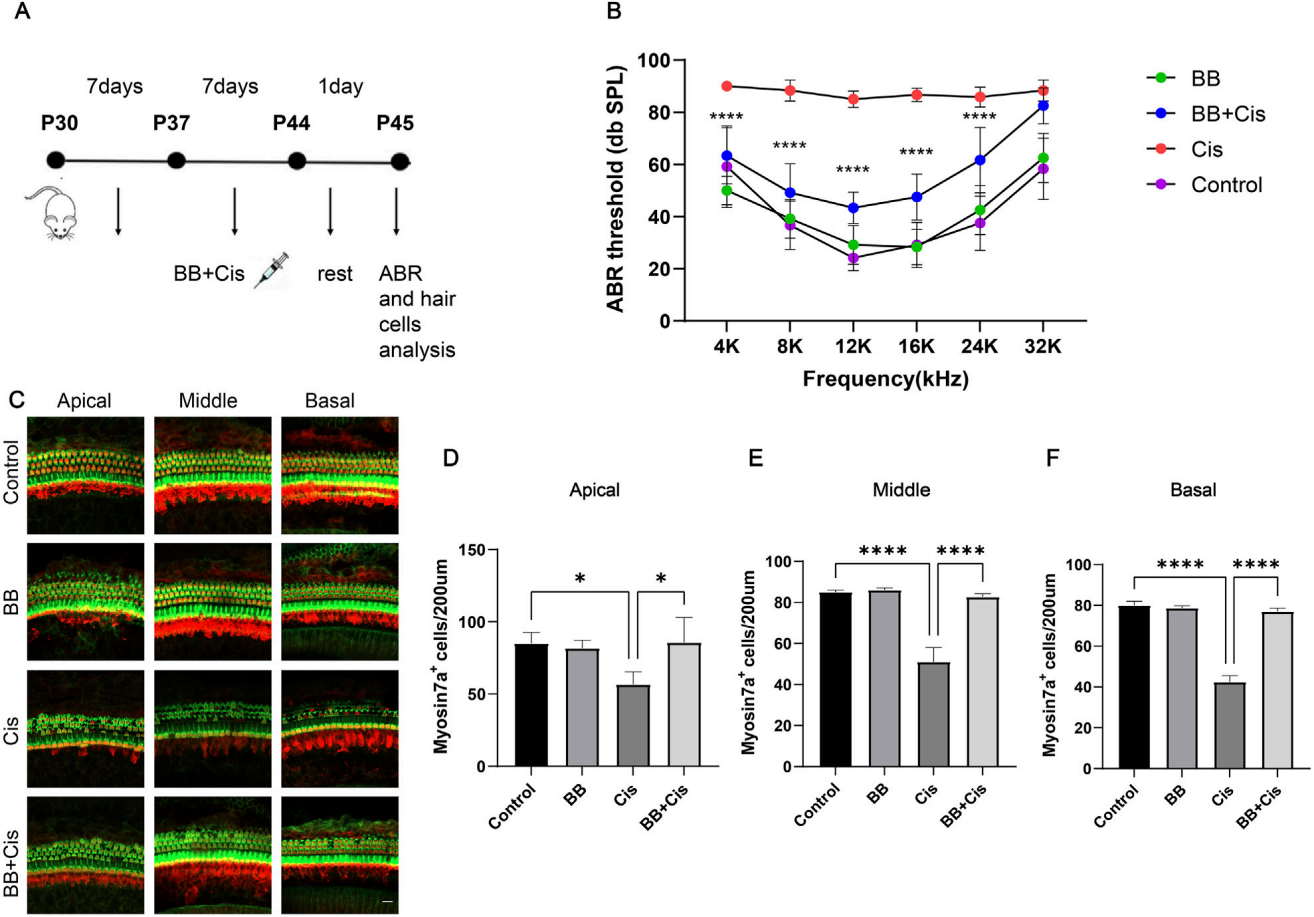
BB shield mice from hearing impairment caused by cisplatin exposure. **(A)** Schematic of the cisplatin-induced damage model. **(B)** ABR threshold analysis showing BB’s effect on cisplatin-induced hearing loss in C57 mice (n = 6). **(C)** Myosin 7a immunofluorescence visualizes cochlear hair cells across the apex, midsection, and base of the cochlea. Red fluorescence: myosin7a Green fluorescence: Phalloidin **(D–F)** Numerical analysis of cochlear hair cell populations in the apex, midsection, and base (n = 3). *P < 0.05, ****P < 0.0001. BB: Berberrubine; Cis: Cisplatin.

By creating mouse models to induce hearing loss with cisplatin (Figure 2A), Our investigation delved into the potential of BB to safeguard against hearing impairment triggered by cisplatin. The ABR results revealed severe hearing impairment in the cisplatin group with significantly elevated thresholds. Conversely, the group treated with BB showed a significant decrease in hearing impairment caused by cisplatin across the tested frequencies, which included 4, 8, 12, 16, and 24 kHz (Figure 2B). The immunofluorescence examination revealed a significant depletion of hair cells across the cochlea apex, midsection, and base in the basilar membrane area. Furthermore, pretreatment with BB mitigated this damage and restored hair cell counts, underscoring its role in safeguarding cochlear hair cells (Figures 2C–F). In conclusion, BB exhibits a protective effect against hearing loss caused by cisplatin in mice, highlighting its potential as a therapeutic agent for mitigating the ototoxic effects of cisplatin.

### 3.3 BB reduces apoptosis of HEI-OC1 cells triggered by cisplatin

**FIGURE 3:**
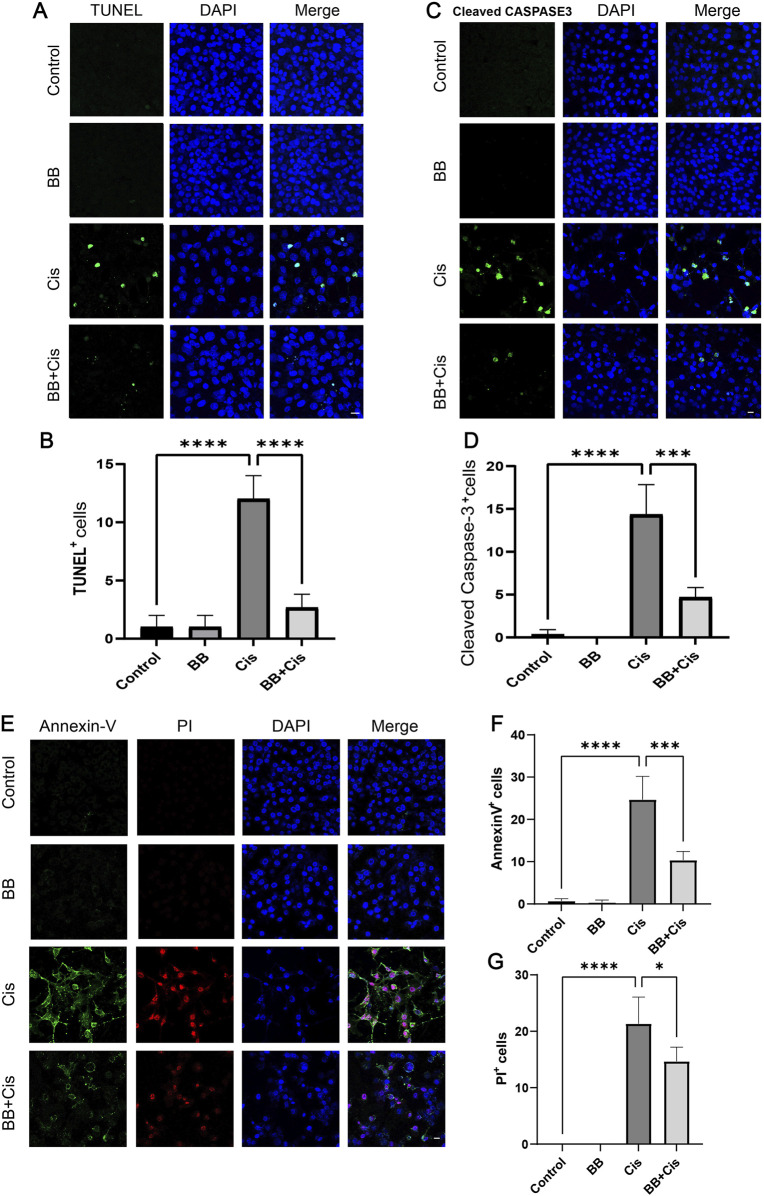
BB reduces apoptosis of HEI-OC1 cells triggered by cisplatin. **(A)** TUNEL assay shows BB’s influence on apoptosis in cisplatin-treated HEI-OC1 cells. Scale bar: 20 μm. **(B)** Quantification of TUNEL-positive cells in **(A)** (n = 3). **(C)** Cleaved Caspase-3 immunofluorescence indicates BB’s modulation of the apoptotic response to cisplatin. Scale bar: 20 μm. **(D)** Numerical count of Cleaved Caspase-3-positive cells in **(C)** (n = 3). **(E)** Annexin V-PI staining highlights BB’s effect on apoptosis initiated by cisplatin. Scale bar: 20 μm. **(F, G)** Quantification of Annexin V-positive and PI-positive cells in **(E)** (n = 3). *P < 0.05, ***P < 0.001, ****P < 0.0001. BB: Berberrubine; Cis: Cisplatin.

We utilized TUNEL and Cleaved Caspase-3 staining to evaluate apoptosis, pretreated HEI-OC1 cells with BB for 12 h before exposing them to cisplatin for 24 h. Compared to the control group, the group treated with cisplatin showed a significant elevation in indicators of apoptosis, such as TUNEL positivity and levels of Cleaved Caspase-3. In contrast, the group pretreated with BB showed a notable decrease in these apoptotic markers (Figures 3A–D). Additionally, the use of Annexin V-PI staining provided an additional method to evaluate the extent of apoptosis. Under the same treatment conditions, cisplatin exposure resulted in higher apoptosis rates than the control group. However, the addition of BB substantially reduced the apoptotic markers (Figures 3E–G). In summary, BB significantly mitigates cisplatin-induced apoptosis in HEI-OC1 cells.

### 3.4 BB mitigates the apoptosis of hair cells in cochlear explants induced by cisplatin

**FIGURE 4:**
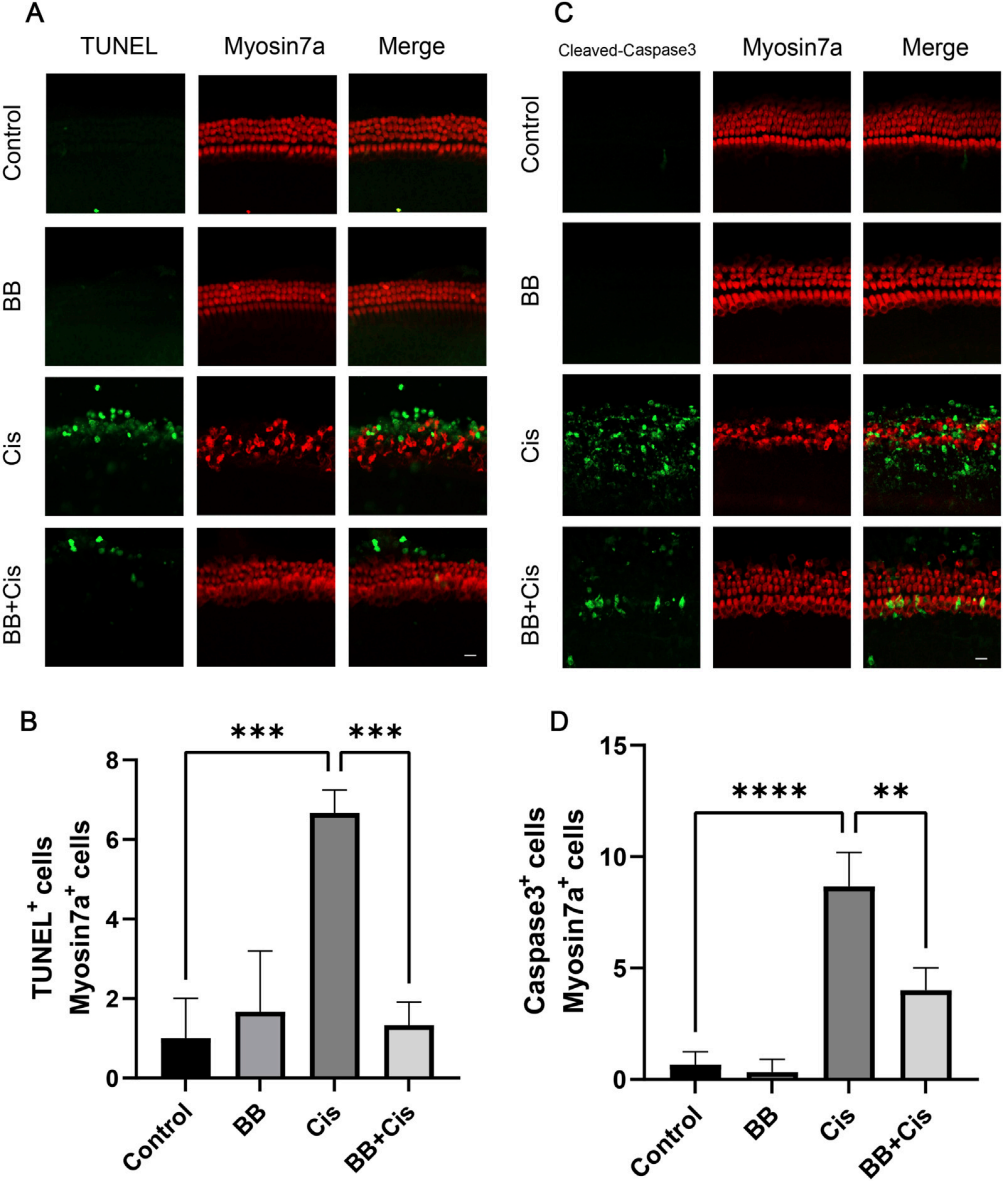
BB mitigates the apoptosis of hair cells in cochlear explants induced by cisplatin. **(A)** Midsection cochlear hair cells were pre-treated with BB and then exposed to cisplatin, followed by TUNEL staining. Scale bar: 20 μm. **(B)** Count of TUNEL and myosin 7a double-positive cells in **(A)** (n = 3). **(C)** Cochlear hair cells in the middle turn were stained for Cleaved Caspase-3 after BB pre-treatment and cisplatin exposure. Scale bar: 20 μm. **(D)** Numerical analysis of Cleaved Caspase-3 and myosin 7a double-positive cells in **(C)** (n = 3). **P < 0.01, ***P < 0.001, ****P < 0.0001. BB: Berberrubine; Cis: Cisplatin.

In our study, cochlear explants underwent TUNEL and Cleaved Caspase-3 staining. BB was introduced at a concentration of 5 µM for 12 h, followed by cisplatin exposure for 24 h. Immunofluorescence analysis revealed that, in the cisplatin-treated group, the count of dual positive cells for TUNEL/myosin 7a and Cleaved Caspase-3/myosin 7a was elevated. Conversely, BB treatment mitigated hair cell damage, substantially reducing the levels of TUNEL and Cleaved Caspase-3, indicative of apoptosis (Figures 4A–D). In conclusion, BB significantly reduced hair cell apoptosis induced by cisplatin in the cochlear basement membrane in cochlear explants.

### 3.5 BB reduces cisplatin-induced oxidative stress response

**FIGURE 5:**
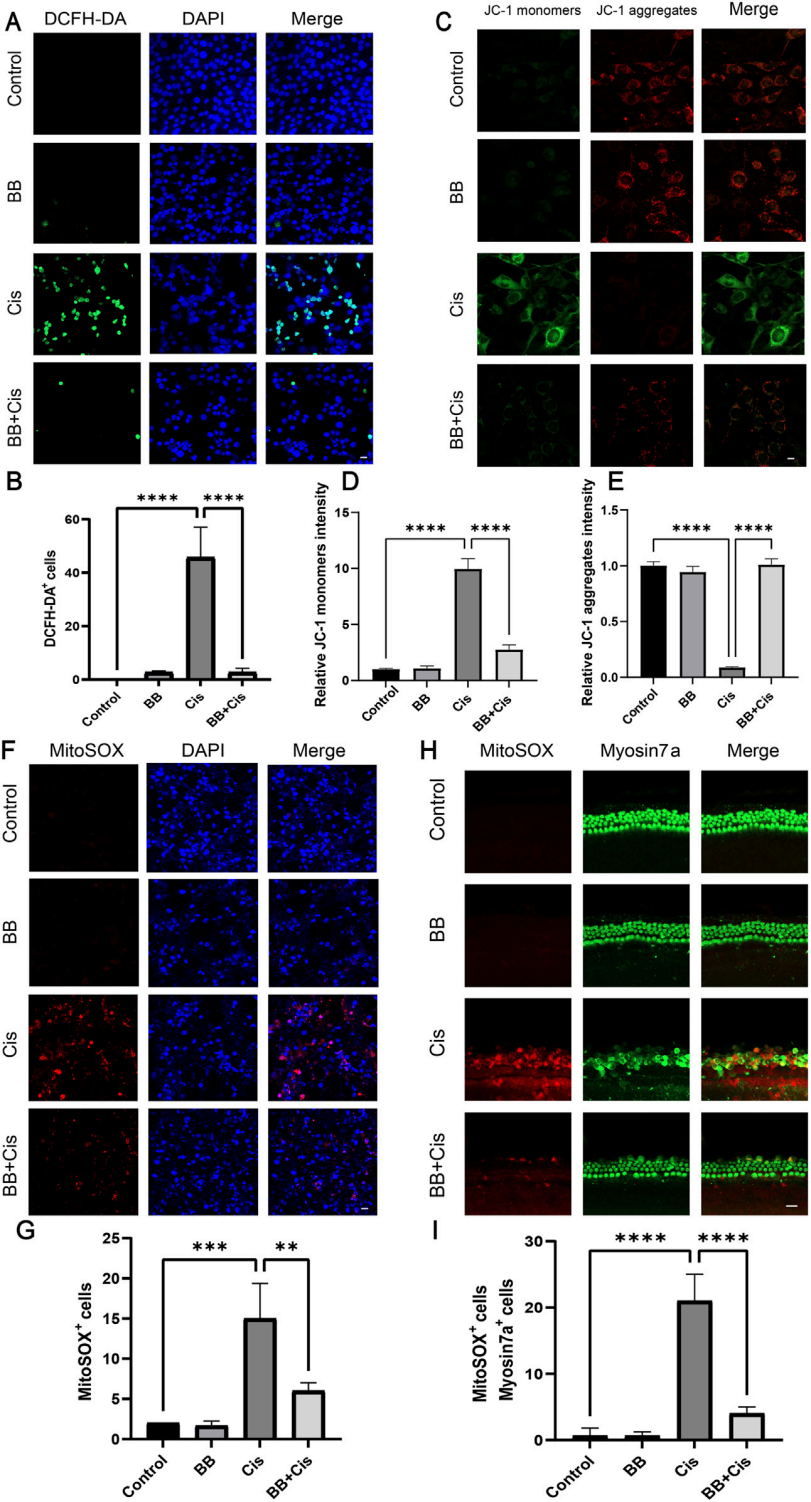
BB reduces cisplatin-induced oxidative stress response. **(A)** DCFH-DA fluorescence labeling of different experimental groups. Scale bar: 20 μm. **(B)** Numerical assessment of fluorescence intensity in **(A)**. **(C)** JC-1 staining of HEI-OC1 cells. Scale bar: 20 μm. **(D, E)** Numerical evaluation of JC-1 fluorescence in **(C)**. **(F)** MitoSOX staining for mitochondrial superoxide detection. Scale bar: 20 μm. **(G)** Numerical analysis of MitoSOX fluorescence in **(F)**. **(H)** MitoSOX staining in cochlea’s middle turns from various groups. Scale bar: 20 μm. **(I)** Numerical evaluation of data in **(H)**. **P < 0.01, ***P < 0.001, ****P < 0.0001. BB: Berberrubine; Cis: Cisplatin.

A significant accumulation of ROS in the mitochondrial compartment is crucial for the onset of hearing loss caused by cisplatin. To investigate ROS levels in HEI-OC1 cells, we utilized DCFH, MitoSOX staining, and JC-1 analysis to assess mitochondrial membrane potential. Following treatment with BB and cisplatin, the cells were stained with DCFH, MitoSOX, and JC-1. Following cisplatin treatment, a noticeable rise in the number of cells staining positively for DCFH, MitoSOX, and JC-1 indicators was detected when compared with the untreated control group. Notably, pre-treatment with BB significantly decreased the number of cells showing positivity for DCFH, MitoSOX, and JC-1 in HEI-OC1 cells (Figures 5A–G). Furthermore, the expression of MitoSOX in cochlear explants was evaluated in a similar fashion. In line with the cell-based results, cisplatin administration led to an elevation in ROS levels in cochlear hair cells, while BB pre-treatment mitigated ROS production (Figures 5H–I). In summary, the findings demonstrate that BB successfully diminishes the production of ROS triggered by cisplatin in both the HEI-OC1 cell line and cochlear explant cultures.

### 3.6 Promoting folate synthesis may be one of the mechanisms by which berberine antagonizes cisplatin ototoxicity

**FIGURE 6:**
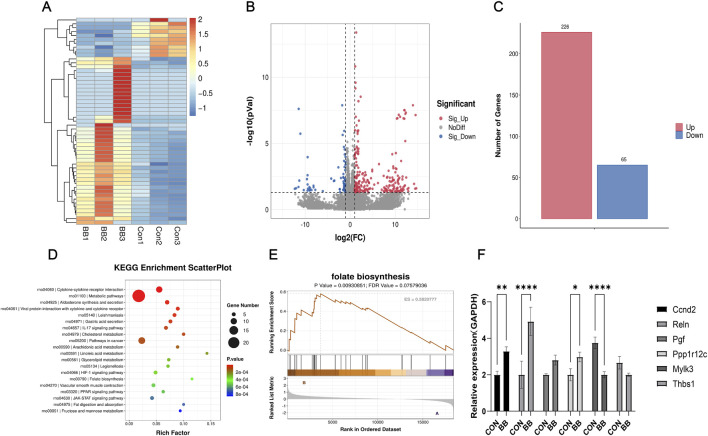
BB antagonizes cisplatin ototoxicity by promoting folate synthesis. **(A)** Heat map of genes with substantial expression changes post BB treatment. **(B)** Scatter plot of genes showing notable variance in expression post BB treatment. **(C)** Analysis of upregulated and downregulated gene quantities post BB treatment. **(D)** KEGG pathway enrichment of the most notably altered pathways post BB treatment. **(E)** Gene Set Enrichment Analysis (GSEA) contrasting gene expression in folate biosynthesis pathways between control and BB-treated groups. **(F)** qRT-PCR evaluation of gene expression levels in the folate biosynthesis pathway (n = 3). Statistical significance: *P < 0.05, **P < 0.01, ****P < 0.0001. BB: Berberrubine; Cis: Cisplatin.

To further investigate how BB antagonizes cisplatin-induced ototoxicity, an RNA sequencing analysis was conducted on HEI-OC1 cells following treatment with BB. The data indicated that treatment with BB led to the upregulation of 226 genes and the downregulation of 65 genes, in contrast to the gene expression levels observed in the control group (Figures 6A–C). Pathway enrichment analysis based on the Kyoto Encyclopedia of Genes and Genomes (KEGG) database identified that one of the major pathways affected by BB treatment is the promotion of folate synthesis (Figure 6D). Following up with Gene Set Enrichment Analysis (GSEA), it was found that the pathway associated with folate biosynthesis was significantly enriched in the group treated with BB when compared to the untreated control group (Figure 6E). Notably, genes involved in folate biosynthesis and metabolism such as Ccnd2, Reln, Pgf, Mylk3, Ppplr12c, and Thbsl were significantly altered in the BB treatment group. Real-time fluorescence quantitative PCR further validated the changes in mRNA expression related to folate biosynthesis in the BB treatment group compared to the control group (Figure 6F). The expression of Ccnd2, Reln, Pgf, and Ppplr12 genes increased, while the expression of Mylk3 and Thbsl genes decreased. The overall effect of these changes is to promote folate synthesis. The collective results imply that Promoting folate synthesis may be one of the mechanisms by which berberine antagonizes cisplatin ototoxicity.

## 4 Discussion

Although cisplatin is a prevalent chemotherapy drug for malignancies, its associated ototoxicity poses a significant issue, affecting patients’ quality of life and potentially necessitating the use of hearing aids or other supportive measures. Oxidative stress, primarily from overproduction of Reactive oxygen species (ROS) are central to cisplatin-induced ototoxicity, undermining the cochlea’s natural defenses (Ramkumar et al., 2021; Tan et al., 2022; Li et al., 2023b). Cisplatin also provokes an inflammatory response characterized by the release of cytokines TNF-α, IL-1β, and IL-6 (Ramkumar et al., 2021; Zhang et al., 2020; Qiao et al., 2024). This response is amplified by heightened levels of COX-2, iNOS, and TNF-α, which are stimulated by ROS through STAT1 activation and NOX3 enzyme induction (Altun et al., 2014; Estfanous et al., 2020; Martins et al., 2017; Shalkami et al., 2018; Umugire et al., 2023; Yin et al., 2023). Furthermore, cisplatin’s interaction with DNA leads to cytotoxic cross-linking, triggering apoptosis through increased Bax expression, mitochondrial permeability changes, and the activation of caspase-9 and caspase-3 (He et al., 2022; Umugire et al., 2023; Sheth et al., 2017; Wang et al., 2023). The complexity of these mechanisms underscores the need for continued research into effective strategies to combat cisplatin ototoxicity.

In our research, we exhibited the protective capabilities of BB against cisplatin-induced hearing damage and investigated the potential mechanisms behind it. Our findings indicated that BB reduces the detrimental effects on hair cells and the incidence of apoptosis induced by cisplatin, as observed in both the HEI-OC1 cell line cultures and the cochlear explants *in vitro*. Furthermore, BB was shown to attenuate the accumulation of reactive oxygen species (ROS) and the damage to mitochondria within these cells. To further authenticate BB’s defense against cisplatin-induced hearing damage, we conducted mouse experiments using a cisplatin-induced injury model. In our study, oral administration of BB to mice effectively protected them from cisplatin-induced hearing loss, as evidenced by reduced hearing thresholds across a range of frequencies. However, this protective effect was not observed at the 32 kHz frequency. The lack of protection at this high frequency may be due to the greater severity of cisplatin’s damage at higher frequencies, which could result in irreversible harm that BB is unable to counteract. Our immunofluorescence tests confirmed BB’s protective role on hair cells in the cochlea’s basal turn basement membrane. Nonetheless, the intricate physiological environment of animals could allow cisplatin to affect other unidentified critical structures. These unidentified effects might limit BB’s ability to prevent high-frequency hearing loss, even when hair cell survival is improved. In summary, while BB demonstrates promise in mitigating cisplatin-induced ototoxicity, its protective effects are not absolute, particularly at the highest sound frequencies. Further research is needed to understand the full scope of cisplatin’s impact on the auditory system and to explore additional strategies that may enhance BB’s protective capabilities.

To further investigate the protective mechanisms of BB against cisplatin-induced hearing loss, we conducted RNA sequencing analysis. This analysis revealed that a total of 226 genes were upregulated and 65 genes were downregulated in HEI-OC1 cells following BB treatment. KEGG and GSEA analysis showed significant enrichment of genes promoting folate synthesis pathway. Genes such as Ccnd2, Reln, Pgf, Mylk3, Ppplr12c, and Thbsl showed significant changes in expression. The overall effect of these gene expression changes is to upregulate the folate synthesis signaling pathway. Our experimental findings suggest that BB facilitates folate biosynthesis in organisms. Folate, a vital vitamin in the human body, possesses a wide array of physiological functions. Various studies have reported that folate contributes to the development of the nervous system, metabolism, epilepsy and depression management, cardiovascular health, pregnancy-related conditions, and cancer prevention (Balashova et al., 2018; Liwinski and Lang, 2023; Otsu et al., 2023; Ponziani et al., 2012; Shulpekova et al., 2021; Yang et al., 2012). A multitude of sources assert that folic acid plays a significant role in the prevention and management of hearing impairments. This includes sensorineural hearing loss that occurs with aging and cases of sudden sensorineural hearing loss with neurological origins (Kabagambe et al., 2018; Kose Celebi et al., 2023; Kundu et al., 2012). The protective mechanisms likely involve mitigating oxidative stress responses, inhibiting apoptosis, and promoting angiogenesis in the inner ear (Martínez-Vega et al., 2015; Martínez-Vega et al., 2016; Uchida et al., 2011). Additionally, there is clear evidence in the literatures stating that folate can prevent and treat cisplatin-induced ototoxicity (Tanyeli et al., 2019). Animal studies have corroborated this, aligning with our research findings. Therefore, it is probable that BB’s antagonistic effect on cisplatin ototoxicity is mediated by enhancing the biosynthesis of folate substances.

Our study has several limitations. Firstly, we could have further optimized the selection of cisplatin and BB concentrations and doses. We selected these concentrations in cell experiments using different concentration gradients, which was a scientifically reliable approach. However, in the *in vitro* cochlear explant experiments, we relied on experience and logical deduction, increasing drug concentrations due to the greater tolerance of tissues compared to cells. Although the experiment achieved significant effects, there may still be an optimal drug concentration. For subsequent animal experiments, we referred to existing literatures on BB, but found no studies specifically on the ear. The optimal concentration of BB may vary across tissues, organs, and diseases. Our examination of BB’s toxicity revealed it to be harmless at low and moderate levels but harmful at high levels, emphasizing the importance of careful BB concentration regulation in practical use. Therefore, further optimization of drug concentrations is necessary. Secondly, cisplatin ototoxicity damages a variety of tissues and structures, such as hair cells, vascular striae, spiral ganglia, and synapses (Wang et al., 2023; Gu et al., 2022; Waissbluth et al., 2022). Our research primarily focused on examining how BB can protect hair cells within the organ of Corti. We did not investigate and observe other structures, such as supportive cells, spiral ganglia, and vascular striae, while our findings provide valuable insights into the protective effects of BB on hair cells, they are limited in scope and do not encompass the full range of ototoxic effects of cisplatin on the auditory system. Further research is needed to explore the impact of BB on other affected structures and to gain a more comprehensive understanding of its protective mechanisms. Finally, we identified differentially expressed genes after BB treatment through RNA sequencing and performed qRT-PCR to assess mRNA expression at the cellular level. Our study’s scope was limited, and further validation at different tissue and animal levels is necessary. We can also conduct Western blotting experiments to validate the results at the protein expression level, so that our conclusions will be more scientifically robust and persuasive.

## 5 Conclusion

It effectively showcases the protective benefits of BB against hearing damage induced by cisplatin by mechanisms involving the enhancement of folate biosynthesis, the reduction of ROS generation, and the mitigation of apoptosis. This concept may offer a novel approach to mitigating the ototoxic effects of cisplatin in a clinical setting.

## Data Availability

The original contributions presented in the study are included in the article/supplementary material, further inquiries can be directed to the corresponding author.
